# The Interplay between Dietary Habits and Glycemic Control in Type 1 Diabetes: A Comprehensive Prospective FGM Study

**DOI:** 10.3390/life14091153

**Published:** 2024-09-12

**Authors:** Maja Gradinjan Centner, Daniela Čačić Kenjerić, Ema Schönberger, Hrvoje Centner, Daria Sladić Rimac, Kristina Steiner, Romana Marušić, Miro Bakula, Daniela Fabris Vitković, Ivica Mihaljević, Ines Bilić Ćurčić, Silvija Canecki Varžić

**Affiliations:** 1 Department of Endocrinology, Clinical Hospital Center Osijek, 31000 Osijek, Croatia; majagradinjan@gmail.com (M.G.C.); ema.schonberger7@gmail.com (E.S.); daria.rimac@gmail.com (D.S.R.); steiner.kristina1@gmail.com (K.S.); silvija.canecki@gmail.com (S.C.V.); 2 Faculty of Food Technology Osijek, University J. J. Strossmayer, 31000 Osijek, Croatia; daniela.kenjeric@ptfos.hr; 3 Faculty of Medicine Osijek, University J. J. Strossmayer, 31000 Osijek, Croatia; hcentner@gmail.com (H.C.); romana.marusic55@gmail.com (R.M.); ivanm2712@gmail.com (I.M.); 4 Department of Nursing and Palliative Medicine, Faculty of Dental Medicine and Health Osijek, University J. J. Strossmayer, 31000 Osijek, Croatia; 5 National Memorial Hospital “Dr. Juraj Njavro”, 32000 Vukovar, Croatia; 6 Division of Endocrinology, Diabetes and Metabolic Diseases, Department of Internal Medicine, Sveti Duh University Hospital, 10000 Zagreb, Croatia; mbakula@kbsd.hr; 7 School of Medicine, University of Zagreb, 10000 Zagreb, Croatia; 8 General Hospital Pula, 52000 Pula, Croatia; daniela.fabris.dfv@gmail.com; 9 Faculty of Medicine Pula, 52000 Pula, Croatia; 10 Department of Nuclear Medicine, University Hospital Center Osijek, 31000 Osijek, Croatia; 11 Academy of Medical Sciences of Croatia, 10000 Zagreb, Croatia

**Keywords:** type 1 diabetes, glycemic regulation, lifestyle, dietary patterns

## Abstract

Type 1 diabetes has become prevalent among the adult population, who have increasingly gained access to sensing technology. This study delved into the impact of diet, nutritional status, and the use of a continuous glucose monitoring system (CGM) on glycemic regulation among adults diagnosed with T1D. Employing a prospective design, data were gathered from 151 participants aged 18–60 across multiple cycles. Participants utilized the FreeStyle Libre (FSL) Flash Glucose Monitoring (FGM) System and provided dietary details via questionnaires and diaries. The findings unveiled correlations between dietary patterns and glycemic control, with higher protein intake associated with improved glycated hemoglobin A1C values (*p* = 0.019), yet elevated fat and protein consumption was linked to heightened rates of hyperglycemia. Conversely, no significant relationship was observed between dietary variables and hypoglycemia occurrence. Interestingly, subjects with more readings of glucose levels consumed fewer carbohydrates (*p* = 0.004) and more proteins (*p* = 0.000). Furthermore, physical activity and marital status correlated with glycemic stability, while higher education was associated with enhanced glycemic control (*p* = 0.021). This study confirmed the importance of structured education on glycemic regulation and the importance of dietary patterns in glucose management. Also, the educational role of the FGM system in changing dietary habits was confirmed, which is one of the key factors for improving glycemic regulation in continuous glucose monitoring system users.

## 1. Introduction

Type 1 diabetes mellitus (T1D) arises from the autoimmune destruction of pancreatic beta cells, culminating in the complete depletion of insulin production, but the exact mechanism behind T1D remains unclear [1]. However, it seems that the interplay of genetic, environmental, and immunological factors plays an important role in T1D development. The management of T1D includes insulin replacement therapy, structured patient education, adherence to a balanced diet, and consistent physical activity [2]. Despite comprehensive education and the integration of new technologies, achieving optimal self-management and regulation of diabetes remains challenging for many patients. Studies conducted worldwide have revealed that a considerable proportion of patients fail to attain target glycated hemoglobin (A1C) levels, which typically range from 8 to 9% [3,4].

Self-monitoring of plasma glucose levels can be carried out using two methods. The first method involves using a glucometer, which often causes discomfort and anxiety in patients due to the need for multiple finger pricks to obtain capillary blood samples. This discomfort can lead to reluctance in testing, thereby limiting the detection of hypoglycemic episodes [5,6]. Another modern method for self-monitoring glycemic values is through continuous glucose monitoring (CGM) devices. In Croatia, the “FreeStyle Libre (FSL) Flash Glucose Monitoring (FGM) System” has been available since August 2018. A CGM system represents a significant advancement in the management of T1D. By providing real-time information on glucose levels, as well as the direction and rate of glucose fluctuations, CGM empowers patients to actively participate in their disease management. By alerting users to impending low blood glucose levels, CGM systems enable timely interventions to mitigate or prevent hypoglycemia, reducing the risk of associated complications and enhancing overall safety and well-being [7,8].

Dietary recommendations for individuals with diabetes are similar to guidelines for the general population, with some slight modifications. These adjustments help individuals with diabetes manage their blood glucose levels more effectively by providing uniform carbohydrate intake within each food group. For instance, starchy vegetables such as potatoes, beans, and peas are classified within the bread and substitutes group rather than the vegetable group. Moreover, cheese is categorized within the meat and substitutes group, rather than the dairy group. Therefore, modern guidelines increasingly emphasize individual education and personalized dietary recommendations [9,10]. The diet based on carbohydrate units, also known as the free diet, is characterized by its flexible approach. In this method, one carbohydrate unit (CU) typically contains 15 g of carbohydrates. This approach is particularly beneficial for individuals with T1D, as it empowers them to make informed food choices by learning how to read food labels and understand the composition of different foods [11,12].

A more recent approach regarding diabetic education suggests dividing meals into three main meals, with incorporated snacks [13,14]. This approach offers the advantage of more structured meal planning, allowing for individuals to focus on balanced nutrition during their main meals while still incorporating snacks as needed. Research indicates that skipping breakfast and/or having a late dinner is associated with higher A1C values and lower odds of achieving good glycemic control. Furthermore, increasing the number of meals is linked to greater variability in blood glucose levels [15,16].

Available research indicates that total energy intake does not directly impact the regulation of postprandial glucose values. Instead, these values are influenced by the composition of the meal itself [17]. Therefore, it is suggested that when adjusting insulin doses to optimize postprandial glucose values, consideration should be given to the entire meal composition, not just carbohydrates [18]. It has been established that the glycemic index (GI) of a meal directly influences postprandial glucose values, insulin response, and c-peptide levels. Consequently, the general recommendation is to prioritize foods with low to medium GI [19]. Indeed, not all carbohydrates are created equal, and attention to dietary fiber intake is crucial. Several studies suggest that increasing dietary fiber intake, typically recommended at around 30 to 35 g per day, can confer significant benefits [20,21].

Lately, the proportion of micronutrients in the diet is increasingly recognized for its role in glycemic regulation. In numerous clinical trials, the intake of zinc, with a recommended daily allowance (RDA) of 15 mg/day, has been monitored either through dietary sources or supplementation. Higher zinc intake has been associated with better regulation of fasting glucose, A1C, and insulin levels. Similarly, studies investigating selenium intake, with an RDA of 55 μg/day, highlight improvements in glucose levels, insulin sensitivity, reduction of insulin resistance, decreased blood lipids, and certain inflammatory markers [22,23]. Furthermore, adequate magnesium intake has been linked to better A1C values and improved lipid profiles in individuals with T1D [24,25].

This research aims to provide new perspectives on the influence of diet on regulation of T1D and conversely the influence of FSL FGM technology on dietary habits potentially improving glycemic control and mitigating the risk of hypoglycemic episodes.

## 2. Materials and Methods

This study included 151 patients with T1D who use the FSL FGM system and were treated in the outpatient clinic at the Department of Endocrinology, University Hospital Centre Osijek, Croatia in the period from 15 September 2019 to 30 November 2021. The recruitment of subjects was based on inclusion and exclusion criteria shown in Table 1.

The study protocol required seven visits to the outpatient clinic at the Department of Endocrinology, University Hospital Centre Osijek, further described in more detail.

During the recruitment period, the study protocol was explained to each subject and written consent was obtained. Every subject completed all surveys and food diary. During the study, there were no subjects’ withdrawals.

### 2.1. Research Design and Methods

**Figure 1:**
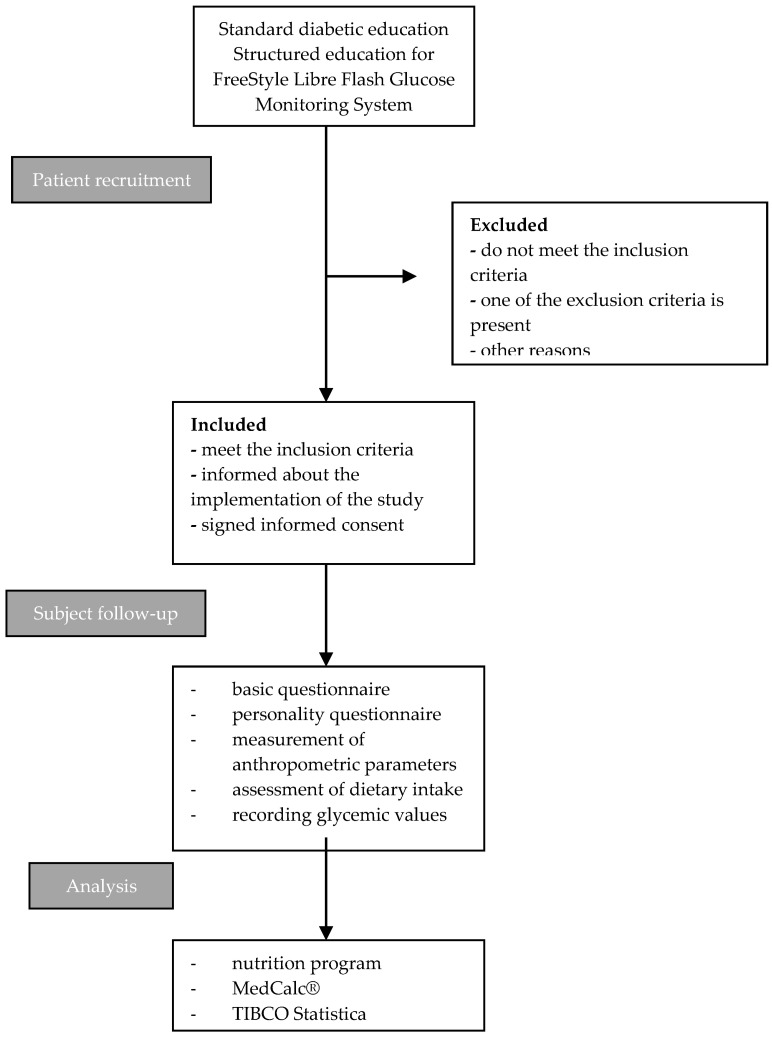
Systematic review of study protocol.

This was a prospective observational study, conducted in accordance with the Helsinki Declaration, and a positive opinion was issued by the Ethics Committee of the University Hospital Centre Osijek on 12 September 2019 (R2-12487/2019). The study protocol is shown in detail in Figure 1.

During the initial visit, all patients underwent standard diabetic education which included principles of diabetic diet and nutrition based on carbohydrate units. Relevant medical history data were collected using a specifically designed questionnaire according to the study design. In addition, the respondents also self-assessed physical activity frequency (less than 20 min per day; light activity several times per week—i.e., walking, cycling; intensive activity daily—minimum 45 min per day) and use of nutritional supplements. Depending on the answer they were divided into two groups. Patients who had less than 20 min of physical activity per day were categorized as physically inactive, and the rest were categorized as physically active. This categorization was made based on American Diabetes Association guidelines, which recommend at least 150 min per week of moderate-intensity exercise [26].

During the first visit, an anthropometric measurement was performed using the Tanita analyzer body composition DC-360, Amsterdam, The Netherlands. The measurement included body mass, BMI, BMR, total fat tissue, total muscle tissue, bone mass, and visceral adipose tissue. Body mass was measured for all subjects during the research on the same site-standardized scale, a Seca 704S class III, Hamburg, Germany, to the nearest 0.1 kg, in underwear, without shoes, after the subject had been fasting for a period of at least 10 h. Body height was measured on a standardized height meter to the nearest 0.5 cm.

For each subject, data on dietary habits were collected using the diet diary form. The form included general information about the subject and data on food intake presented in a table that included days of the week; time of consumption; and types and quantities of consumed food, drinks, and nutritional supplements. During the first visit, the subjects filled in the first day of the food diary. During the next 5 visits or contacts (by e-mail or telephone) the subjects independently filled in the food diary. During the last visit, which was carried out in person due to the analysis of body composition and biochemical blood sampling, the subjects filled in the last day of the food diary.

At the baseline, participants were divided into two groups using an A1C and time in range (TIR)—A1C ≤ 7.0% and TIR ≥ 70% indicated good blood glucose control, whereas A1C > 7.0% and TIR < 70% indicated poor glycemic control. Parameters of glycemic control accepted by international consensus guidelines for interpreting continuous glucose monitoring (mean glucose, glucose management indicator (GMI), percentage of time in target range (TIR, glucose values 3.9–10.0 mmol/L), percentage of time above the target range (TAR, glucose values > 10.1 mmol/L), percentage of time below target range (TBR, glucose values < 3.9 mmol/L), scanning frequency, percentage of captured FGM data, and time spent in hypoglycemia) were analyzed using reports generated from the FSL FGM system (Abbott, Chicago, IL, USA) [27].

To achieve optimal efficiency of the FSL system, each subject was individually trained on the principles of operation and usage of the system. CGM metric data were collected during the second and the last visit.

### 2.2. Statistical Analysis

The sample size calculation was made assuming a test power of 0.8 for significance threshold 0.05 to perform independent samples *t*-test (effect size 0.5), ANOVA test (effect size 0.25) and correlation coefficient (effect size 0.6) (G*Power software, version 3.1.9.4., University of Dusseldorf, Dusseldorf, Germany). Data from food diary were analyzed using Nutrition Program, IG PROG, Rijeka, Croatia (IG PROG, 2021). Categorical data were represented by absolute and relative frequencies. Numerical data were presented as the arithmetic mean and standard deviation in the case of the normal distributions while in other cases the median and interquartile range were used. Differences in categorical variables were tested with the chi-square test and, if necessary, with Fisher’s exact test. The normality of the distribution of numerical variables was tested by Shapiro–Wilk test. The differences of normally distributed numerical variables between two independent groups were tested with Student’s *t* test and in case of deviation from normal distribution with a Mann–Whitney U test. The correlation of normally distributed numerical variables was assessed by Pearson’s test by the correlation coefficient r, and in case of deviation from the normal distribution by Spearman’s correlation coefficient ρ (rho). All *p* values are two-sided. The significance level was set to α = 0.05. Statistical analysis was performed using MedCalc^®^ Statistical Software version 20.010 (MedCalc Software Ltd., Ostend, Belgium, 2021) and TIBCO Statistica, Version 14.0. (Statistics, Palo Alto, CA, USA, 2015).

## 3. Results

This study included 151 participants aged 18–60 years. Most participants, 127 (84.1%), used MDI, while 23 (15.2%) were treated with an insulin pump. The majority of respondents, 56.3%, had stable body weight within a year. The baseline characteristics of the subjects are presented in Table 2.

Body weight was higher in men, 78.5 kg [interquartile range (IQR) 70.2–87.7] versus 78.5 kg (IQR 70.2–87.7) in women. Analysis of body mass composition showed that women had a higher proportion of total adipose tissue with 19.6% (IQR 14.9–23.2), while men had a higher proportion of muscle mass 59.9% (IQR 56.1–66.9) (Table 3).

No differences were observed in the relationship between glycemic regulation and body mass. Subjects who did not have changes in body mass spent the least time in TBR (*p* = 0.030) (Table 4).

Subjects living within the marital union spent significantly more time in normoglycemia (*p* = 0.021), had lower average glucose values (*p* = 0.060) as well as less frequent incidence of hyperglycemia (*p* = 0.067) and hypoglycemia (*p* = 0.056) (Table 5).

Single people consumed more fat (*p* = 0.030), but also had a higher intake of foods rich in zinc (*p* = 0.025) and magnesium (*p* = 0.029), which can be associated with possible supplementation. In addition, differences were observed at the level of 10% significance, meaning that people who were married consumed more protein (*p* = 0.100) (Table 6).

The relationship between nutritional variables and baseline A1C was assessed utilizing Spearman’s rank correlation coefficient. An inverse, i.e., negative, correlation between baseline A1C and average daily fibrous foods intake was observed (Rho = −0.2590; *p* = 0.015) (Table 7).

No significant correlation was observed between average consumption of individual macronutrients and baseline glycemic regulation (Table 8).

After 3 months a significant negative correlation was observed between the initial A1C and the average daily protein intake (Rho = −0.296; *p* = 0.012) meaning that people who consume more protein-rich foods have lower A1C values (Table 9).

At the initial placement of the FSL system, all subjects were educated about the importance of proper nutrition and maintaining good glycemic regulation, and after three months, significantly better glycemic regulation (*p* = 0.022) was noted (Table 10).

Furthermore, significantly better glycemic regulation after 3 months was observed in subjects whose diet contained more protein (*p* = 0.019) and selenium (*p* = 0.027) (Table 11).

A significantly positive correlation between time spent in normoglycemia and average daily protein intake (Rho = 0.249; *p* = 0.032) was noticed using the Spearman correlation test (Table 12).

No significant correlation was found between dietary parameters and hypoglycemia (Table 13).

The Spearman test examined the correlations between dietary variables and the number of daily measurements. Subjects who consumed more protein (Rho = 0.489; *p* = 0.000) and fewer carbohydrates (Rho = −0.336; *p* = 0.004) had a significantly higher scanning frequency. Likewise, a positive correlation was observed between the scanning frequency and fiber intake; subjects who consumed more fiber had higher scanning frequency. In addition, there was a positive correlation between scanning frequencies and the average intake of selenium (Rho = 0.277; *p* = 0.019) (Table 14).

Physically active subjects had a higher TIR at the level of 10% significance (*p* = 0.100), (Table 15).

## 4. Discussion

Our findings highlight the significant impact of dietary choices on glycemic management, with a protein-rich diet proving to be particularly effective. Participants with higher daily protein intake did not only achieve target A1C values more successfully, but also spent significantly more time in normoglycemia, indicating a strong positive correlation between protein consumption and improved glycemic control. Previous studies have shown a beneficial effect of high-protein diets on glycemic regulation. In a study conducted on T1D patients, a daily protein intake of >1.2 g/kg was associated with a 6.9% higher TIR and an 8% reduced TBR [28]. Proteins modulate glycemia through multiple physiological mechanisms. They stimulate the pancreatic β-cells to secrete insulin, thereby enhancing glucose uptake by peripheral tissues and reducing plasma glucose levels. Simultaneously, increased protein intake can elevate glucagon levels, which acts antagonistically to insulin by promoting glycogenolysis and gluconeogenesis in the liver, thus supporting blood glucose homeostasis. The ingestion of dietary proteins also slows gastric emptying and delays the absorption of carbohydrates, resulting in a mitigated postprandial glycemic response. Additionally, amino acids from protein digestion can be utilized for gluconeogenesis, especially during fasting or low carbohydrate availability, contributing to the maintenance of blood glucose levels [29]. On the other hand, a meta-analysis including 1138 T2D patients did not show a significant impact of high-protein diets on glycemic regulation, but it did demonstrate a reduction in low-density lipoprotein (LDL) cholesterol, total cholesterol, triglycerides, and HOMA-IR levels in T2D patients, which is certainly a reduction in cardiovascular risk, crucial for these patients. This emphasizes the need to assess both the immediate and long-term effects of high-protein diets on overall health, including their impact on cardiovascular risk factors [30].

Furthermore, our study reveals that individuals with better A1C values tended to consume a diet rich in dietary fiber, aligning with previous research suggesting a significant role of dietary fiber intake in glycemic control [31,32]. One study found that higher dietary fiber intake significantly improves glycemic control in adults with prediabetes and diabetes, reducing A1C by 2.00 mmol/mol, along with improvements in cardiometabolic health markers such as total cholesterol, LDL cholesterol, and BMI [32]. Dietary fiber improves postprandial hyperglycemia by slowing the digestion and absorption of carbohydrates and increasing satiety, which leads to weight loss. In individuals with insulin resistance, dietary fiber may enhance peripheral insulin sensitivity, possibly through short-chain fatty acids produced by the fermentation of fiber in the intestines. Additionally, consuming dietary fiber can reduce total and LDL cholesterol levels, help regulate blood pressure, accelerate the movement of food through the digestive system, and balance intestinal pH, stimulating fermentation in the intestines and leading to the production of short-chain fatty acids [33,34].

Also, those achieving target GMI values had significantly higher selenium levels in their diet, which was also shown in recent studies [22,23]. Selenium supplementation may enhance glycemic regulation by reducing reactive oxygen species (ROS) and upregulating the activity of selenoproteins, such as glutathione peroxidases, which are critical in mitigating oxidative damage and improving insulin sensitivity. One meta-analysis found that selenium supplementation significantly reduces insulin levels, HOMA-IR, and increases HDL cholesterol levels in patients with cardiometabolic diseases. However, it showed no significant effect on fasting plasma glucose or other lipid profile markers [35]. Other meta-analysis showed that selenium supplementation has a positive effect on fasting insulin, but no effect on A1C, HOMA-IR, or fasting blood sugar [36]. Further research, particularly well-designed randomized controlled trials with larger and more diverse populations, is needed to clarify the role of selenium in glycemic control and to determine optimal dosages for potential therapeutic use.

In addition, individuals with higher daily scanning frequency consumed more protein-rich foods and fewer carbohydrates. Furthermore, individuals who monitor their blood glucose levels more frequently tend to have higher selenium intake in their diet. These findings may be associated with the fact that individuals who prioritize proper nutrition and regular physical activity generally achieve better glycemic values, as confirmed by previous researches [30,37,38,39]. Our study also confirmed that participants engaging in physical activity spent a higher percentage of time in normoglycemia underscoring the importance of regular exercise in maintaining stable blood glucose levels. Numerous studies have shown reductions in glycated hemoglobin, fasting blood glucose, BMI, and waist circumference as a result of the exercise intervention [40]. A 2–10% reduction in body weight over 1–4 years is accompanied by decreases in A1C ranging from 0.2–1.0%. Activities that help reduce glucose levels include taking breaks during extended periods of sitting, planning workouts after meals to prevent spikes in blood sugar, and incorporating both aerobic and high-intensity resistance exercises into weekly routine. Several studies have confirmed that achieving 150 min per week of moderate to vigorous physical activity, spread across 3–5 days (including both aerobic and resistance exercises), is effective in reducing cardiometabolic risk factors [41].

However, in our study cohort, 25% of male and 16.48% of female participants exhibited increased body mass, while 8.33% of males and 16.48% of females were classified as obese. Gender disparities in body composition were anticipated and validated among the participants of this study. Women exhibit a higher proportion of total body fat, whereas men tend to have greater muscle mass and visceral fat, as substantiated by prior studies [42]. It is worth noting that the categorization of participants based on fat tissue composition reveals a distinct pattern compared to BMI-based categorization, pointing out the necessity for careful selection of assessment tools. In our study, gender differences were more pronounced in males, particularly regarding normal nutritional status and increased body mass, suggesting the limited sensitivity of BMI alone.

When considering glycemic regulation and changes in body mass, it was shown that participants who did not experience changes in body mass recorded the least amount of time spent in hypoglycemia. This finding is consistent with numerous studies highlighting the fact that in individuals with T1D stability in body mass is associated with less pronounced fluctuations in glycemic profile [43,44]. Such results underscore the importance of continuous monitoring and individually tailored approaches in diabetes management, emphasizing how maintaining a constant body mass can contribute to better hypoglycemia control and overall disease management. Moreover, studies demonstrated that carb counting contributes to better A1C control, with minimal changes in body weight [45,46].

The association between dietary variables and the occurrence of hypoglycemia was also explored, revealing that these parameters are not correlated with the prevalence of hypoglycemia. This may be attributed to the fact that participants underwent uniform education on nutrition, therapy, and physical activity, enabling them to anticipate hypoglycemic episodes using the FSL FGM system. Additionally, more frequent hypoglycemic episodes are observed in children and adolescents [47] and older adults [48]. However, this study focused on individuals of middle age due to potential confounding factors in younger and older age groups contributing to hypoglycemia. These findings corroborate previous research highlighting the importance of adhering to dietary guidelines to manage T1D [49].

We also analyzed the association between glycemic parameters and marital status. In our study, it has been demonstrated that individuals who are in some form of marital union spend significantly more time in normoglycemia compared to single people. Differences at the 10% significance level were observed, indicating that individuals living in a marital union exhibit better average glucose values and fewer occurrences of hyperglycemia and hypoglycemia. Upon examining the dietary patterns of participants in this study regarding marital status, it was noticed that singles consume more fatty foods. Many authors suggest that individuals in a marital or partnership union pay more attention to the quality of their diet than singles. Individuals in a marital union are more likely to prepare and consume meals together, using fewer pre-packaged and semi-prepared products [50]. Married individuals often benefit from shared responsibilities and emotional support, which can lead to better adherence to diabetes management routines, including diet and medication compliance. Additionally, the social and emotional support provided by a spouse has been associated with lower stress levels, which can positively impact glycemic control by reducing the physiological effects of stress on blood sugar levels. However, the quality of the marital relationship plays a crucial role, as high levels of marital stress or conflict may negate these benefits and even worsen glycemic outcomes [51,52].

Furthermore, all participants received education on the significance of proper nutrition and maintaining adequate glycemic control. A comparison of A1C values before and three months after education revealed significant positive differences, indicating improved regulation post-education. This underscores the hypothesis of numerous authors regarding the crucial role of thorough and regular education for patients and their family members [37,53]. The emphasis on education not only aligns with the findings of improved glycemic control, but also with the observed correlation between higher protein consumption and increased time spent in normoglycemia among study participants [30,37,38].

Overall, while our study contributes valuable insights into the complex interplay between body composition, nutritional status, and glycemic regulation in individuals with T1D, further research is warranted to fully elucidate these relationships and inform tailored interventions for optimal diabetes management. There are some limitations to our study such as lack of diversity in ethnicity, wide age range, disproportionate sex distribution, and potential selection bias as only users of continuous glucose monitoring were included. Also, during the follow-up period, A1C was not assessed; instead, initial A1C values were compared with GMI. Although GMI provides a good estimate of glycated hemoglobin based on sensor values, discrepancies between these two indicators can often occur within the same subject. Some studies indicate that despite advancements and the widespread use of CGM systems, A1C continues to be a crucial parameter for assessing glycemic control [54].

Future directions for this study could involve expanding to a multicenter approach, incorporating multiple clinical centers to enhance the diversity and generalizability of the findings. Increasing the sample size would provide more robust data and improve the statistical power of the study. Extending the follow-up period would allow for a more comprehensive understanding of the long-term effects on glycemic control. Narrowing the age range of participants could help control for various factors that influence glycemic regulation, leading to more precise results. Additionally, including a more diverse population in terms of ethnicity and socioeconomic status, especially considering the impact of socioeconomic factors on dietary choices, would offer deeper insights into the broader applicability of the findings.

## 5. Conclusions

Participants consuming a diet rich in protein achieved target A1C values more frequently. In addition, participants with higher energy intake, consuming more fats and protein, exhibited a higher incidence of hyperglycemia. Those achieving the highest proportion of normoglycemia consumed adequate protein intake alongside recommended energy levels. Still, no significant relationship was found between dietary variables and the occurrence of hypoglycemia probably due to structured education and CGM usage. Also, those who were physically active tended to spend a higher percentage of time in normoglycemia, as did married people. Participants who maintained a stable body weight for a year, as measured at the beginning and end of the study, spent the least amount of time experiencing hypoglycemia. At the end of the study period, subjects with a higher frequency of daily scanning consumed more protein-rich foods and fewer simple carbohydrates emphasizing the importance of the CGM system itself as an educational tool in identifying appropriate food choices as well as their impact on glycemia. Our findings support the usage of the CGM system in all patients regardless of the type of diabetes or therapeutic modality as an educational tool for changing lifestyle and achieving better disease management.

## Figures and Tables

**Table 1:** Inclusion and exclusion criteria in the recruitment of patients.

Inclusion Criteria	Exclusion Criteria
Subjects diagnosed with type 1 diabetes mellitus.	Type 2 diabetes mellitus or other specific form of diabetes.
Established indication for the use of the “FreeStyle Libre Flash Glucose Monitoring System” according to national insurer criteria.	Use of medications with a significant impact on glycemic status.
Insulin therapy—multiple daily injections or SAP * insulin pump.	Duration of illness < 12 months.
Subjects older than 18 and younger than 60.	Acute infectious diseases.
Preserved cognitive and psychomotor abilities.	Pregnancy.
Until the moment of inclusion in this study, subjects did not use the continuous glucose monitoring system.	Planned surgical interventions.
	Confirmed allergy to medical adhesives prior to or during this study.
	Disorders of sensory organs.
	Active psychiatric treatment.

* SAP, sensor-augmented pump.

**Table 2:** Baseline characteristics of subjects participating in this study.

Variable	Median (IQR)	Minimum–Maximum
Age (years)	38 (28.0–50.0)	(18.0–60.0)
Gender		*n* (%)
Female Male		91 (60.3) 60 (39.7)
Insulin administration		*n* (%)
Insulin pump		23 (15.2)
MDI		127 (84.1)
Marriage status		*n* (%)
Married		88 (58.3)
Unmarried		63 (41.7)
Physical activity frequency		*n* (%)
Less than 20 min per day		17 (11.3)
Light activity several times per week (walking, bicycling)		121 (80.1)
Intensive activity daily (minimum 45 min per day)		13 (8.6)
Changes in body weight over the past year		*n* (%)
None		85 (56.3)
Weight gain		27 (17.9)
Weight lost		39 (25.8)
Nutritional supplements		*n* (%)
Yes		102 (67.5)
No		49 (32.5)

Multiple daily injections (MDI).

**Table 3:** Body mass composition depending on gender.

Gender	Median (IQR)	Minimum–Maximum
Female weight (kg)	63.8 (57.1–72.7)	42.4–113.2
Male weight (kg)	78.5 (70.2–87.7)	43.6–128.3
Female height (cm)	166.0 (160–170)	150.0–186
Male height (cm)	181.5 (176–185)	163–203
Female body mass index (kg/m^2^)	22.9 (21–26.4)	15.0–41.6
Male body mass index (kg/m^2^)	24.0 (21.9–26.4)	16.4–36.3
Muscles tissue in female (%)	41.7 (38.6–47)	28.5–103
Muscles tissue in male (%)	59.9 (56.1–66.9)	35.2–102
Fat tissue in female (%)	19.6 (14.9–23.2)	8.8–55.6
Fat tissue in male (%)	16.6 (10.4–21.1)	2.8–47
Bone in female (%)	2.2 (2.1–2.5)	1.6–3.1
Bone in male (%)	3.1 (3–3.5)	1.9–4
Visceral tissue in female (%)	5.0 (2–7)	1–14
Visceral tissue in male (%)	7 (2–11)	1–19
Basal metabolic rate in female (kcal)	1333 (1233.5–1489)	992–1870
Basal metabolic rate in male (kcal)	1840 (1699–2095)	1146–2494

**Table 4:** Differences between glycemic parameters related to changes in body weight.

	No Changes in Body Weight	Weight Loss	Weight Gain	
FGM Parameters	Mean (SD)	Mean (SD)	Mean (SD)	p *
Mean glucose (mmol/L)	9.1 (1.9)	8.6 (2.14)	9.5 (3.07)	0.514
GMI (%)	7.3 (1.21)	7.3 (1.86)	7.4 (2.7)	0.753
TAR (%)	35.5 (18.91)	34.2 (26.78)	41.2 (28.99)	0.703
TIR (%)	57.8 (18.31)	59.9 (25.94)	52.8 (29.42)	0.786
TBR (%)	6.5 (5.89)	6.0 (5.12)	5.9 (5.55)	0.901
Hypoglycemic events (*n*)	39.6 (38.51)	42.9 (60.28)	31.7 (40.72)	0.406
The average duration of hypoglycemic events (min)	89.1 (30.33)	110.3 (26.86)	95.8 (54.4)	0.030
FGM data capture (%)	85.9 (15.02)	92.4 (11.66)	84.4 (25.27)	0.138
Scanning frequency (*n*)	13.7 (13.52)	13.2 (4.87)	12.2 (5.57)	0.472

* Kruskal–Wallis test. Flash glucose monitoring (FGM), glucose management indicator (GMI), time above target range (TAR), time in target range (TIR), and time below target range (TBR).

**Table 5:** Differences in glycemic control among married and unmarried subjects.

	Unmarried	Married	
FGM Parameters	Mean (SD)	Mean (SD)	p *
Mean glucose (mmol/L)	9.68 (2.6)	8.75 (1.9)	0.060
GMI (%)	7.69 (1.6)	7.13 (1.8)	0.143
TAR (%)	42.3 (23.3)	33.1 (22.8)	0.067
TIR (%)	50 (22.7)	61.4 (22.2)	0.021
TBR (%)	7.7 (6.8)	5.5 (4.6)	0.056
Hypoglycemic events (*n*)	36 (35.1)	40.1 (49.8)	0.695
The average duration of hypoglycemic events (min)	98.7 (44.4)	94 (32.4)	0.592
FGM data capture (%)	86.3 (16.8)	87.6 (17.4)	0.753
Scanning frequency (*n*)	14.3 (16.3)	12.7 (5.2)	0.507

* *t*-test. Flash glucose monitoring (FGM), glucose management indicator (GMI), time above target range (TAR), time in target range (TIR), and time below target range (TBR).

**Table 6:** Differences between nutritional variables and marriage status.

	Married	Unmarried	
Average Daily Intake	Mean (SD)	Mean (SD)	p *
Energy (kcal)	2042.8 (479.1)	1928.8 (291.9)	0.120
Carbohydrates (g)	197.4 (63.8)	211.1 (49.2)	0.207
Proteins (g)	121.3 (49.6)	109 (28.4)	0.100
Fats (g)	85.8 (41.7)	72.6 (21.9)	0.030
Fiber (g)	21.2 (7.7)	22.7 (7.4)	0.337
Sugar (g)	63 (38.1)	60.8 (28.9)	0.733
Zinc (mg)	13.3 (4.1)	11.7 (2.9)	0.025
Selenium (µg)	51.9 (12.3)	49.7 (9.7)	0.311
Magnesium (g)	0.32 (0.1)	0.27 (0.1)	0.029

* *t*-test.

**Table 7:** Correlation of nutritional variables and baseline A1C.

	Spearman’s Rank Correlation Coefficient (*p*-Value)
Nutritional Variables (Average Daily Intake)	Baseline A1c
Energy (kcal)	−0.0200 (0.854)
Proteins (g)	−0.0932 (0.390)
Carbohydrates (g)	−0.1519 (0.160)
Fats (g)	0.1167 (0.282)
Fiber (g)	−0.2590 (0.015)
Sugar (g)	0.0092 (0.932)

Glycated hemoglobin (A1C).

**Table 8:** Correlation of macronutrients and baseline A1C.

Spearman’s Rank Correlation Coefficient (*p*-Value)	
Macronutrients (%)	Baseline A1c
Proteins	−0.121 (0.365)
Fats	−0.065 (0.625)
Carbohydrates	0.099 (0.457)

Glycated hemoglobin (A1C).

**Table 9:** Correlation of nutritional variables and baseline A1C after 3 months.

	Spearman’s Rank Correlation Coefficient (*p*-Value)
Average Daily Intake	Baseline A1c
Energy (kcal)	−0.187 (0.117)
Proteins (g)	−0.296 (0.012)
Carbohydrates (g)	0.129 (0.282)
Fats (g)	−0.169 (0.155)
Fiber (g)	−0.087 (0.470)
Sugar (g)	−0.035 (0.773)

Glycated hemoglobin (A1C).

**Table 10:** Differences between baseline A1C and GMI.

	Mean (SD)	Difference (SD)	p *
Baseline A1c	7.881 (1.413)	0.378 (1.379)	0.022
GMI	7.503 (1.596)		

* *t*-test. Glycated hemoglobin (A1C); glucose management indicator (GMI).

**Table 11:** Differences between glycemic control and nutritional variables after 3 months.

	Good Glycemic Control	Poor Glycemic Control	
Average Daily Intake	Mean (SD)	Mean (SD)	*p* *
Energy (kcal)	2046.8 (438.3)	1901.4 (363.2)	0.127
Carbohydrates (g)	194.6 (48.6)	200.4 (49.6)	0.619
Proteins (g)	126.3 (48.9)	104.1 (29.9)	0.019
Fats (g)	84.9 (44.1)	76.2 (22.8)	0.272
Fiber (g)	23.3 (7.23)	21.9 (7.86)	0.415
Sugar (g)	55.6 (24.5)	62.3 (39.9)	0.416
Zinc (mg)	13.1 (3.40)	12.3 (3.47)	0.347
Selenium (µg)	55.4 (9.91)	50.3 (9.29)	0.027
Magnesium (g)	0.29 (0.11)	0.30 (0.10)	0.701

* *t*-test.

**Table 12:** Correlation between nutritional variables and TIR.

	Spearman’s Rank Correlation Coefficient (*p*-Value)
Nutritional Variables	TIR
Energy (kcal)	0.223 (0.057)
Proteins (g)	0.249 (0.032)
Carbohydrates (g)	−0.074 (0.529)
Fats (g)	0.211 (0.072)
Fiber (g)	0.124 (0.293)
Sugar (g)	0.041 (0.729)

Time in target range (TIR).

**Table 13:** Correlation between nutritional variables and TBR.

	Spearman’s Rank Correlation Coefficient (*p*-Value)
Nutritional Variables	TBR
Energy (kcal)	−0.063 (0.606)
Proteins (g)	−0.096 (0.434)
Carbohydrates (g)	0.185 (0.069)
Fats (g)	−0.119 (0.328)
Fiber (g)	0.069 (0.573)
Sugar (g)	0.217 (0.074)

Time below target range (TBR).

**Table 14:** Correlation between nutritional variables and scanning frequency.

	Spearman’s Rank Correlation Coefficient (*p*-Value)
Nutritional Variables	Scanning Frequency
Energy (kcal)	0.187 (0.115)
Proteins (g)	0.489 (0.000)
Carbohydrates (g)	−0.336 (0.004)
Fats (g)	0.206 (0.083)
Fiber (g)	0.232 (0.049)
Sugar (g)	−0.089 (0.458)
Zinc (mg)	0.214 (0.071)
Selenium (µg)	0.277 (0.019)
Magnesium (g)	0.208 (0.080)

**Table 15:** Differences between FGM parameters depending on physical activity.

FGM Parameters	Physical Active	Physical Inactive	
	Average (SD)	Average (SD)	p *
Mean glucose (mmol/L)	8.86 (2.0)	10.03 (2.1)	0.103
GMI (%)	7.20 (1.7)	7.91 (1.4)	0.246
TAR (%)	34.1 (21.9)	46.8 (22.1)	0.106
TIR (%)	59.5 (21.4)	46.8 (24.2)	0.100
TBR (%)	6.3 (5.6)	6.4 (7.1)	0.935
Hypoglycemic events (*n*)	42.8 (48.4)	22.1 (21.0)	0.237
The average duration of hypoglycemic events (min)	94.2 (34.1)	112.6 (53.4)	0.183
FGM data capture (%)	87.4 (17.4)	79.0 (20.4)	0.232
Scanning frequency (*n*)	13.9 (11.2)	8.1 (2.8)	0.152

* *t*-test. Flash glucose monitoring (FGM), glucose management indicator (GMI), time above target range (TAR), time in target range (TIR), and time below target range (TBR).

## Data Availability

The data presented in this study are available on request from the corresponding author.
